# Efficacy and Safety of AbobotulinumtoxinA for the Treatment of Hemiparesis in Adults with Lower Limb Spasticity Previously Treated With Other Botulinum Toxins: A Secondary Analysis of a Randomized Controlled Trial

**DOI:** 10.1002/pmrj.12348

**Published:** 2020-03-27

**Authors:** Alberto Esquenazi, Gaëtan Stoquart, Peter Hedera, Luis Jorge Jacinto, Ugo Dimanico, Francois Constant‐Boyer, Allison Brashear, Anne‐Sophie Grandoulier, Claire Vilain, Philippe Picaut, Jean‐Michel Gracies

**Affiliations:** ^1^ Department of Physical Medicine and Rehabilitation MossRehab Gait and Motion Analysis Laboratory Elkins Park PA USA; ^2^ Physical and Rehabilitation Medicine Department, Cliniques universitaires Saint‐Luc Catholic University of Louvain Brussels Belgium; ^3^ Department of Neurology, Division of Movement Disorders Vanderbilt University Nashville TN USA; ^4^ Centro de Medicina de Reabilitação de Alcoitão, Estoril Estoril Portugal; ^5^ Division of Physical Medicine and Rehabilitation, Department of Surgical Sciences University of Turin Turin Italy; ^6^ Unités de Médecine Physique et de Réadaptation, Hôpital Sébastopol Université de Reims Champagne‐Ardenne Reims France; ^7^ School of Medicine University of California Davis Sacramento CA; ^8^ Biostatistics Ipsen Pharma Les Ulis France; ^9^ Medical Affairs Les Ulis France; ^10^ Service de Rééducation Neurolocomotrice EA 7377 BIOTN, Université Paris‐Est, Hospital Albert Chenevier‐Henri Mondor Créteil France; ^11^ Department of Neurology Wake Forest School of Medicine Winston‐Salem NC

## Abstract

**Objective:**

To examine the safety and efficacy of abobotulinumtoxinA in patients previously treated with botulinum toxin type A (BoNT‐A) products other than abobotulinumtoxinA.

**Design:**

Secondary analysis from a phase 3, double‐blind, single‐cycle, randomized, placebo‐controlled study.

**Setting:**

Fifty‐two centers (11 countries).

**Patients:**

Adults with spastic hemiparesis were randomized (1:1:1) to receive abobotulinumtoxinA 1000 U, 1500 U, or placebo in their affected lower limb.

**Main Outcome Measurements:**

Muscle tone (6‐point Modified Ashworth Scale [MAS], 0‐5) for the gastrocnemius‐soleus complex (GSC); proportion of MAS responders (≥1‐point improvement); angle of catch (X_V3_) and spasticity grade (Y) for the GSC and soleus. Assessments were at weeks 1, 4, and 12 post‐injection. Only descriptive statistics are presented.

**Results:**

Of 388 patients, 84 received previous BoNT‐A treatment (abobotulinumtoxinA 1000 U: N = 30; abobotulinumtoxinA 1500 U: N = 28; placebo: N = 26). At week 4, mean (SD) changes in MAS score in the GSC were − 0.8 (1.1), −0.9 (1.0), and − 0.4 (0.7) for abobotulinumtoxinA 1000 U, 1500 U, and placebo, respectively. Greater MAS responder rates were observed for abobotulinumtoxinA versus placebo at all time points. Mean (SD) changes (week 4) for abobotulinumtoxinA 1000 U, 1500 U, and placebo for X_V3_ were: GSC, 8° (21), 6° (10) and 1° (7); soleus, 11° (21), 5° (9) and 0° (8), respectively; for Y: GSC, −0.4 (0.7), −0.6 (0.8) and − 0.0 (0.9); soleus, −0.5 (0.7), −0.5 (0.7) and − 0.1 (0.6), respectively. Safety data and adverse events were consistent with the overall known profile of abobotulinumtoxinA.

**Conclusions:**

Patients previously treated with other BoNT‐As showed improved muscle tone and spasticity at week 4 following abobotulinumtoxinA injection versus placebo. These findings suggest that abobotulinumtoxinA, at the recommended doses, has a good safety and efficacy profile in adults with lower limb spasticity who were previously treated with other BoNT‐A products.

## Introduction

Several neurological disorders may give rise to lower limb spasticity (LLS) including stroke and brain/spinal cord injury, cerebral palsy, and multiple sclerosis.^1^ AbobotulinumtoxinA (Dysport; Ipsen Pharma, Wrexham, UK), is effective at reducing muscle tone and improving spasticity and functional outcomes in adults with LLS, as shown in several randomized, double‐blind studies,^2^, ^3^ and is licensed in the United States and Europe for the treatment of LLS.^4^, ^5^

In chronic conditions, such as spastic paresis, which require repeat treatment over a prolonged period, immunogenicity (ie, the development of antibodies to the therapeutic product) is a potential concern.^6^ Although the rate of neutralizing antibodies is low across botulinum toxin type A (BoNT‐A) products, secondary nonresponse may occur, which may require the patient to be treated with an alternative BoNT‐A product.^6^ Alternatively, physicians or patients may wish to alter treatment plans based on response to treatment or adverse events, or due to changes in formulary or insurance coverage.^7^ However, there are limited data on whether efficacy and dosing requirements of BoNT‐A products differ from the label recommendations in patients with LLS who have received previous treatment with other BoNT‐A products.

A recent phase 3, double‐blind, single‐cycle study and the open‐label multiple‐cycle extension demonstrated improved muscle tone after a single abobotulinumtoxinA injection to the affected lower limb in adults with LLS following a stroke or traumatic brain injury.^3^ Repeated administration was associated with improved walking speed and likelihood of achieving community ambulation, and abobotulinumtoxinA treatment was well tolerated.^3^ In this double‐blind lower limb study, randomization was stratified according to previous treatment status resulting in two subgroups: not previously treated (naïve) patients, who had not received any previous BoNT‐A injections to the affected lower limb; and previously treated (non‐naïve) patients, who had received at least one previous injection of any BoNT‐A product to the affected lower limb. The aim of the present post hoc analyses of the double‐blind lower limb study was to assess the safety and efficacy of a single cycle of abobotulinumtoxinA in patients who had been previously treated with any BoNT‐A product except for abobotulinumtoxinA.

## Methods

### *Double‐Blind Lower Limb Study Design*

The methodology and results of the phase 3, multicenter, double‐blind, randomized, placebo‐controlled, single‐cycle study have been reported previously.^3^ In brief, patients were randomized 1:1:1 to receive a single injection of abobotulinumtoxinA 1000 U, 1500 U, or placebo. Randomization was stratified according to previous treatment status: not previously treated and previously treated patients, as defined in the preceding text. The total injection volume (7.5 mL) was made up of three vials of either 2.5 mL placebo or reconstituted abobotulinumtoxinA (500 U in 2.5 mL sodium chloride [0.9%]). The content of each 2.5‐mL vial was then pulled out into a single 10‐mL syringe. Patients randomized to abobotulinumtoxinA 1500 U received three vials of abobotulinumtoxinA, those in the 1000 U treatment arm received two vials of abobotulinumtoxinA and one vial of placebo, and patients in the placebo arm received three vials of placebo. A total volume of 7.5 mL was administered to patients by intramuscular injection including 2.5 mL into the soleus muscle (minimum of three sites), 1.5 mL into the medial and/or lateral gastrocnemius muscle (minimum of two sites), and the remainder of the dose injected into at least one other lower limb muscle selected by the investigator from the following additional distal (tibialis posterior, flexor digitorum longus, flexor digitorum brevis, flexor hallucis longus, flexor hallucis brevis) or proximal (rectus femoris, hamstrings, adductor magnus, gracilis, or gluteus maximus) lower limb muscles. Distal and proximal lower extremity muscles were selected based on their potential role in gait dynamics in paretic patients.^8^, ^9^, ^10^ Concomitant medications, such as pain medication, anticholinergic drugs, and skeletal muscle relaxants, were kept stable throughout the study. As the protocol defined, the dose, duration and frequency of treatment was left to the clinical discretion of the investigators. Community‐based physiotherapy initiated at least 1 month prior to the study entry was continued at the same frequency and intensity until week 4 (or study end if possible). Patients were followed‐up for a minimum of 12 weeks and a maximum of 24 weeks.

### *Patients*

Eligible patients were ambulatory adults aged 18 to 80 years with spastic hemiparesis causing gait dysfunction; a comfortable barefoot walking speed of 0.1 to 0.8 m/s, measured on a 10‐meter walking test without walking aids; one clinically defined stroke episode or brain trauma ≥6 months before enrollment; toxin‐naïve and a Modified Ashworth Scale (MAS) score ≥ 2 in the affected gastrocnemius‐soleus complex (GSC) (knee extended), or toxin non‐naïve and a MAS score ≥ 3 in the affected GSC (knee extended) at least 4 months after the last injection of BoNT‐A in the affected lower limb; and GSC spasticity angle ≥5° (Tardieu scale^11^; knee extended).

Patients were excluded if they had major limitation in passive range of motion at the affected hip, knee, or ankle; a known sensitivity to BoNT‐A or abobotulinumtoxinA excipients; severe cognitive impairment that hindered consent provision; or were pregnant. Patients were also excluded if they had received any BoNT‐A injections within 4 months of enrollment.

### *Post hoc Analyses in Previously Treated Patients*

These post hoc analyses included all patients who had previously been treated with any BoNT‐A products except for abobotulinumtoxinA to the affected lower limb at study initiation.

Multiple efficacy end points from the double‐blind study were included in the present analysis. The primary end point of the double‐blind study was change from baseline to week 4 in GSC muscle tone (knee extended), assessed using the MAS (6‐point graded scale: 0, 1, 1+, 2, 3, 4; for quantitative analyses, 1+ was considered as 2 and higher numeric scores were incremented to give a MAS range of 0‐5).

Secondary and tertiary outcome measures assessed in this post hoc analysis were: proportion of MAS responders (≥1 grade improvement from baseline) at weeks 1, 4, and 12; muscle tone (MAS) in the soleus muscle (knee flexed) at baseline, weeks 1, 4, and 12; and spasticity grade in the GSC (knee extended) and soleus muscle (knee flexed) assessed using the Tardieu scale (angle of catch [X_V3_] and severity [Y]) at baseline and weeks 4 and 12. Safety findings, including number of treatment‐emergent adverse events (TEAEs), most common TEAEs, and serious adverse events (SAEs) are also reported.

### *Statistical Analysis*

This was a post hoc analysis and the study was not powered to detect statistical significance. Hence, only descriptive statistics are provided, including mean, standard deviation (SD), and range for continuous variables, and absolute frequency and percentage for categorical variables.

Post hoc analyses of efficacy end points were conducted for the intention‐to‐treat (ITT) population, which included all previously treated patients who received study medication and had data for GSC muscle tone at baseline and week 4. Safety data are reported for the safety population, consisting of all previously treated patients who received study medication.

## Results

### *Study Population*

**Figure 1:**
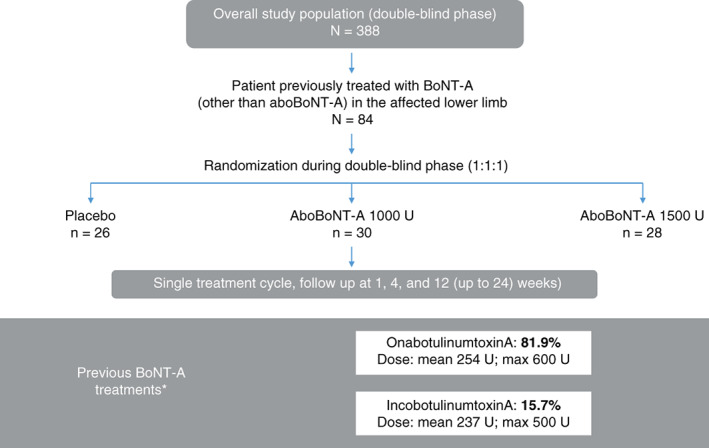
Patient population. AboBoNT‐A, abobotulinumtoxinA; BoNT‐A, botulinum toxin A; max, maximum. *Some patients had previously been treated with more than one other BoNT‐A product. Patient flow is presented for the safety population. Previous BoNT‐A treatments are presented for the intention‐to‐treat population.

A total of 388 patients were enrolled into the double‐blind lower limb study from 52 centers in 11 countries. Of these, 84 had previously received treatment with any BoNT‐A (other than abobotulinumtoxinA) in their affected lower limb and were included in these post hoc analyses. Of the ITT population, 29 patients were randomized to abobotulinumtoxinA 1000 U (safety population: N = 30), 28 patients to abobotulinumtoxinA 1500 U, and 26 patients to placebo (Figure 1).

Baseline demographics and disease characteristics were well matched between treatment arms (Table 1). Mean (SD) age was 52.0 (12.8) years, and the majority (69.9%) of patients were male. Mean (SD; range) time since stroke was 5.7 (5.2; 0.9 to 27.3) years and time since traumatic brain injury was 8.1 (6.1; 1.4 to 21.0) years. The BoNT‐A treatments that patients had received previously were onabotulinumtoxinA (n = 68; 81.9%), incobotulinumtoxinA (n = 13; 15.7%), and other (n = 13; 15.7%). Patients could have received more than one BoNT‐A type. Doses of previous BoNT‐A injections are shown in Table 1. Time from last BoNT‐A injection, other than abobotulinumtoxinA, for treatment of lower limb spasticity to initiating study treatment ranged from 113 to 1769 days, 123 to 2953 days, and 127 to 2359 days, for abobotulinumtoxinA 1000 U, 1500 U, and placebo, respectively. Patients previously treated with abobotulinumtoxinA were not included in this analysis.

**Table 1:** Baseline demographic and disease characteristics in patients previously treated with BoNT‐A (other than aboBoNT‐A)

Parameter	Placebo N = 26	AboBoNT‐A 1000 U N = 29	AboBoNT‐A 1500 U N = 28	All patients N = 83
Age, years	51.1 (14.3) [22‐75]	52.4 (12.1) [26‐73]	52.4 (12.6) [29‐77]	52.0 (12.8) [22‐77]
Male, n (%)	18 (69.2)	22 (75.9)	18 (64.3)	58 (69.9)
BMI, kg/m^2^	26.6 (6.0) [16.2‐43.8]	27.2 (5.5) [20.0‐38.0]	27.9 (4.3) [20.1‐38.3]	27.3 (5.2) [16.2‐43.8]
Affected leg, n (%)				
Left	15 (57.7)	22 (75.9)	12 (42.9)	49 (59.0)
Right	11 (42.3)	7 (24.1)	16 (57.1)	34 (41.0)
Cause of spasticity, n (%)				
Stroke	24 (92.3)	25 (86.2)	25 (89.3)	74 (89.2)
Traumatic brain injury	2 (7.7)	4 (13.8)	3 (10.7)	9 (10.8)
Time since event, years				
Stroke	4.7 (3.1) [1.3‐11.8]	6.4 (7.1) [0.9‐27.3]	5.9 (4.8) [0.9‐19.1]	5.7 (5.2) [0.9‐27.3]
Traumatic brain injury	16.0 (7.0) [11.1‐21.0]	3.8 (2.7) [1.4‐6.5]	8.4 (3.6) [4.4‐11.3]	8.1 (6.1) [1.4‐21.0]
Previous BoNT‐A treatment, n (%)				
OnabotulinumtoxinA	23 (88.5)	21 (72.4)	24 (85.7)	68 (81.9)
IncobotulinumtoxinA	3 (11.5)	5 (17.2)	5 (17.9)	13 (15.7)
Other	2 (7.7)	7 (24.1)	4 (14.3)	13 (15.7)*
Dose of previous BoNT‐A treatment, U				
OnabotulinumtoxinA	254.2 (120.8) [80‐600]	251.6 (103.8) [100‐600]	255.3 (126.8) [50‐600]	253.8 (117.6) [50‐600]
IncobotulinumtoxinA	163.5 (49.7) [100‐200]	221.7 (98.1) [100‐400]	284.5 (123.5) [145‐500]	236.6 (111.1) [100‐500]
Other	275.0 (106.1) [200‐350]	222.5 (121.2) [140‐400]	1733.3 (3329.7) [200‐8500]	986.7 (2377.6) [140‐8500]

BoNT‐A = botulinum toxin A; AboBoNT‐A = abobotulinumtoxinA; BMI = body mass index.*Two patients received Lantox (placebo, n = 1; aboBoNT‐A 1000 U, n = 0; aboBoNT‐A 1500 U, n = 1) and for 11 patients the BoNT‐A type was not specified (placebo, n = 1; aboBoNT‐A 1000 U, n = 7; aboBoNT‐A 1500 U, n = 3). Data are presented for the intention‐to‐treat population and reported as mean (SD) [range] unless otherwise stated.

### *Study Doses Administered*

Overall, the median (range) abobotulinumtoxinA dose administered in the total injection volume was 1000 (867‐1000) U in the 1000 U group and 1500 (800‐1500) U in the 1500 U group (Table 2). Median (range) doses administered by target muscle are detailed in Table 2.

**Table 2:** Treatment doses administered by muscle group

Muscle group	AboBoNT‐A 1000 U N = 30		AboBoNT‐A 1500 U N = 28	
	n	Mean (SD) Median [range] dose, U	n	Mean (SD) Median [range] dose, U
**All sites**	**30**	**993.3 (26.8) 1000 [867‐1000]**	**28**	**1469.3 (132.9) 1500 [800‐1500]**
Lateral gastrocnemius	22	88.8 (29.6) 93 [67‐200]	26	128.8 (25.3) 150 [100‐160]
Medial gastrocnemius	30	141.6 (41.3) 133 [100‐200]	28	169.6 (47.9) 150 [0‐300]
Flexor digitorum longus	23	139.1 (44.6) 133 [67‐267]	23	221.7 (67.1) 200 [100‐300]
Soleus	30	333.3 (0.0) 333 [333‐333]	28	478.6 (95.7) 500 [0‐500]
Tibialis posterior	20	190.0 (92.5) 200 [67‐467]	19	274.7 (111.5) 200 [180‐600]
Flexor digitorum brevis	5	77.3 (31.8) 67 [53‐133]	8	137.5 (74.4) 100 [100‐300]
Flexor hallucis brevis	3	111.1 (38.5) 133 [67‐133]	1	160.0 (−) 160 [160‐160]
Flexor hallucis longus	17	94.9 (33.3) 67 [67‐133]	15	164.0 (47.9) 200 [100‐200]
Adductor magnus	N/A	N/A	1	300.0 (−) 300 [300‐300]
Hamstrings	4	183.3 (63.8) 167 [133‐267]	4	300.0 (81.6) 300 [200‐400]
Rectus femoris	19	186.0 (61.2) 200 [67‐267]	11	372.7 (142.1) 400 [200‐700]
Gluteus maximus	N/A	N/A	N/A	N/A
Gracilis	N/A	N/A	N/A	N/A

AboBoNT‐A, abobotulinumtoxinA; n, number of patients who received an injection in the associated muscle group; N/A, not applicable.

Data are presented for the safety population. The study protocol required that the total injection volume (7.5 mL) was divided between the soleus muscle (exactly 2.5 mL); the medial and/or lateral gastrocnemius muscle (exactly 1.5 mL); and at least one other lower limb muscle selected by the investigator (the remaining injection volume 3.5 mL split between investigator selected sites).

### *Change in GSC (Knee Extended) and Soleus (Knee Flexed) Muscle Scores on the MAS*

At week 4 after a single abobotulinumtoxinA cycle, scores on the MAS were lower than at baseline for both the GSC and the soleus muscle. For the GSC, the mean (SD; range) week 4 changes in score were − 0.8 (1.1; −4 to 0), −0.9 (1.0; −3 to 1) and − 0.4 (0.7; −3 to 0) for abobotulinumtoxinA 1000 U, 1500 U, and placebo, respectively, while for the soleus muscle, the mean (SD; range) week 4 changes in score were −0.7 (1.0; −3 to 1), −0.9 (1.0; −3 to 0), and − 0.5 (0.7; −2 to 1) for abobotulinumtoxinA 1000 U, 1500 U, and placebo, respectively. Data at weeks 1 and 12 are shown in Supplementary Table S1 in Appendix S1.

### *Muscle Tone Responders (≥1 Grade Improvement in MAS Score from Baseline) for GSC (Knee Extended) and Soleus (Knee Flexed) Muscles*

**Figure 2:**
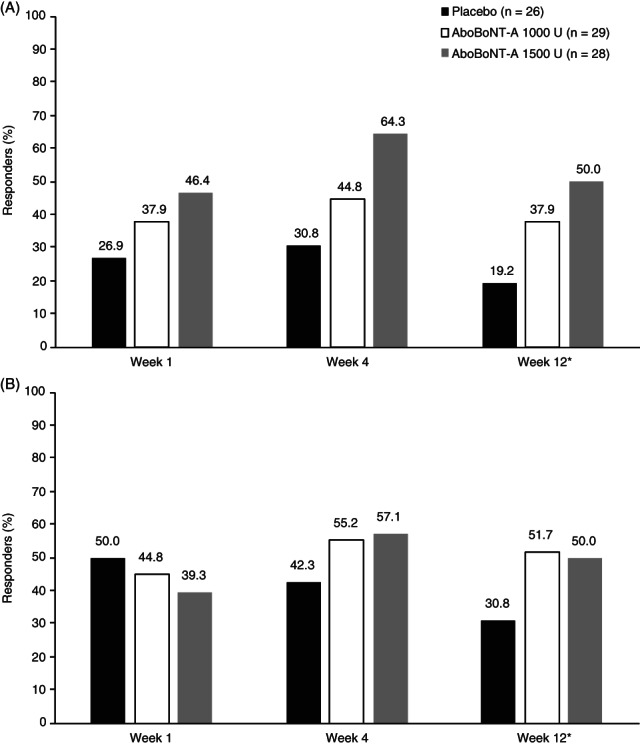
Proportion of responders (≥1 grade improvement) to aboBoNT‐A based on MAS score for (A) GSC muscle tone and (B) soleus muscle tone. AboBoNT‐A, abobotulinumtoxinA; GSC, gastrocnemius‐soleus complex; MAS, Modified Ashworth Scale. *At week 12, n = 1 missing in placebo and aboBoNT‐A 1000 U groups, n = 2 missing in aboBoNT‐A 1500 U group. Data are presented for the intention‐to‐treat population. Responders were defined as patients with at least a one grade improvement in muscle tone (score on the MAS) in comparison to baseline.

For the GSC, a greater proportion of patients responded to treatment with abobotulinumtoxinA than placebo across time points, with a higher responder rate with 1500 U compared with 1000 U abobotulinumtoxinA at each time point (Figure 2A). For the soleus muscle, the proportion of responders increased between weeks 1 and 4 in the abobotulinumtoxinA treatment groups, whereas responder rates in the placebo group decreased from week 1 to week 12 (Figure 2B). For both muscle groups, the highest proportion of responders was observed at week 4 with either abobotulinumtoxinA dose.

### *GSC and Soleus Muscle Spasticity as Measured on the Tardieu Scale*

There were greater improvements in the angle of catch (X_V3_) at week 4 following abobotulinumtoxinA treatment than in the placebo arm for both the GSC and soleus muscle. The mean (SD) change was 8° (21), 6° (10), and 1° (7) for the GSC and 11° (21), 5° (9), and 0° (8) for the soleus muscle with abobotulinumtoxinA 1000 U, abobotulinumtoxinA 1500 U, and placebo, respectively. Spasticity severity (Y) decreased from baseline to week 4 for abobotulinumtoxinA 1000 U and abobotulinumtoxinA 1500 U versus placebo in the GSC (mean [SD]: −0.4 [0.7] and − 0.6 [0.8] vs. −0.0 (0.9), respectively) and soleus muscles (mean [SD]: −0.5 [0.7] and − 0.5 [0.7] vs. −0.1 [0.6], respectively).

### *Safety*

In total, patients previously treated with BoNT‐A experienced 101 TEAEs: 36 (n = 15), 31 (n = 16), and 34 (n = 13) in the abobotulinumtoxinA 1000 U, 1500 U, and placebo groups, respectively (Supplementary Table S2 in Appendix S1). Four patients experienced a TEAE that led to study withdrawal, two in the abobotulinumtoxinA 1000 U group (pancreatic cancer, right hip pain) and one each in the 1500 U group (generalized muscle weakness, considered related to study treatment) and placebo group (loss of consciousness). The most common TEAEs for the abobotulinumtoxinA 1000 U, 1500 U, and placebo groups, respectively, were: fall (13.3%, 10.7%, and 3.8%), muscular weakness (6.7%, 14.3%, and 7.7%), and pain in an extremity (3.3%, 7.1%, and 7.7%). In total, four patients experienced four SAEs; there were two events (sinus tachycardia and pancreatic carcinoma) in the abobotulinumtoxinA 1000 U arm, one event (muscular weakness, considered to be related to study treatment) in the abobotulinumtoxinA 1500 U arm, and one event (loss of consciousness) in patients randomized to placebo.

## Discussion

These post hoc analyses demonstrated that a single cycle of abobotulinumtoxinA administered at either 1000 U or 1500 U reduced muscle tone and improved spasticity grade and angle in adults with spastic hemiparesis of the lower limb that had been treated previously with other BoNT‐A products. Our findings support the primary results for the overall study population and confirm that abobotulinumtoxinA is an efficacious treatment option for adults with LLS.

The improvements in GSC at week 4 we report here in previously treated patients are comparable with those reported for the overall trial population at each abobotulinumtoxinA dose (least squares mean [95% confidence interval]: −0.6 [−0.8, −0.5], −0.8 [−0.9, −0.7], and − 0.5 [−0.7, −0.4] for abobotulinumtoxinA 1000 U, 1500 U, and placebo, respectively).^3^ Similarly, improvements in the angle of catch (X_V3_) and a reduction in spasticity grade were in alignment with those reported in the overall study population (mean [SD] in the GSC and soleus, abobotulinumtoxinA doses combined: X_V3_, +5°^8^ and + 5°,^8^ respectively; Y, −0.3 [0.7] and − 0.4 [0.7], respectively).^3^

Compared with the previously treated patient subgroup, in the overall study population abobotulinumtoxinA dosing was similar across the medial GSC (122.5 U and 183.5 U in the 1000 U and 1500 U groups, respectively), the lateral GSC (95.2 U and 145.6 U in the 1000 U and 1500 U groups, respectively), and the soleus muscle (333.3 U and 495.3 U in the 1000 U and 1500 U groups, respectively), as reported in Table 2.

Our findings demonstrate that recommended doses of abobotulinumtoxinA (1000 U or 1500 U) were well tolerated in this patient population and that safety findings were similar to those experienced by the overall population,^3^ with fall, muscular weakness, and pain in an extremity remaining the most common TEAEs. However, in the non‐naïve population compared with the overall population, slightly higher rates of falls (13.3%, 10.7%, and 3.8% vs. 9.4%, 6.3%, and 3.3%, respectively, in the abobotulinumtoxinA 1000 U, 1500 U, and placebo groups, respectively) and muscular weakness (6.7%, 14.3%, and 7.7% vs 2.4%, 6.3% and 3.1%, in the abobotulinumtoxinA 1000 U, 1500 U and placebo groups, respectively) were observed. Overall, the incidence rate and type of adverse events observed here were also consistent with previous clinical studies of abobotulinumtoxinA^2^ and other BoNT‐A products.^12^ These results suggest that prior treatment with other BoNT‐A products does not affect abobotulinumtoxinA dosing when initiating treatment.

A strength of these analyses was the sourcing of data from a phase 3, randomized, placebo‐controlled study. However, as these analyses were conducted post hoc and were not statistically powered, a limitation is that we report only descriptive statistics here. Furthermore, some secondary end points from the double‐blind lower limb study (such as physician's global assessment and walking speed tests) were not reported here, as it was previously shown that multiple treatment cycles were required to observe meaningful results.^3^ It should be noted that there was a large range among patients in their time since last injection with BoNT‐A's other than abobotulinumtoxinA, as some patients were receiving BoNT‐A treatment at regular intervals prior to this study, while others had not received treatment for a number of years allowing time for contracture, muscle shortening, compensatory gait strategies, and other confounding conditions to develop.

## Conclusion

These post hoc analyses demonstrate that a single cycle of treatment with abobotulinumtoxinA (1000 U or 1500 U) is associated with improvements in both muscle tone and spasticity parameters in adults with LLS previously treated with other BoNT‐A products. The efficacy and safety profile of abobotulinumtoxinA in the previously treated patient subgroup was comparable to the overall study population at similar abobotulinumtoxinA doses. These findings suggest that abobotulinumtoxinA has a good efficacy and safety profile in adults with spastic hemiparesis in the lower limb who have received previous treatment with other BoNT‐A products without the requirement for adjustment to recommended initial dosing.

## Clinical Trial Number

- Phase 3, double‐blind, randomized, placebo‐controlled study: NCT01249404
- Open‐label multiple‐cycle extension: NCT01251367

## Author Contributions

A.E. is the guarantor of this work and, as such, had full access to all the data in the study and takes responsibility for the integrity of the data and the accuracy of the data analysis. Furthermore, individual author contributions included conception and design: A.E., A.B., A.S.G., C.V., P.P. and J.M.G.; acquisition, analysis, and interpretation of data: A.E., G.S., P.H., L.J.J., U.D., F.C.B., A.B., A.S.G., C.V., P.P., and J.M.G.; drafting of the article: A.E., C.V., and P.P.; critical revision for important intellectual content: A.E., G.S., P.H., L.J.J., U.D., F.C.B., A.B., A.S.G., C.V., P.P., and J.M.G.; and final approval of the article: A.E., G.S., P.H., L.J.J., U.D., F.C.B., A.B., A.S.G., C.V., P.P., and J.M.G.

## Author Disclosures

A.E. received research funding from Ipsen and Allergan, and served as a consultant for Merz. G.S. received consultancy fees from Ipsen and Merz. P.H. received royalties from Elsevier publishing; and consultancy fees from Teva and Ipsen. L.J.J. received consultancy fees from Ipsen. F.C.B. received research grants from French Muscular Dystrophy Association; and consultancy fees from Ipsen, Merz, and Medtronic. A.B. received research grants from NINDS, Ipsen, and Revance; and consultancy fees from Ipsen and Revance. A.B. received a consulting fee for other work from Ipsen during the course of this study. During this period, A.B.'s conflict of interest was managed by Wake Forest School of Medicine. A.S.G. is an employee of Atlanstat, subcontracted to Ipsen. C.V. and P.P. are employed by Ipsen. J.M.G. has served as a consultant and received research grant support from Allergan, Ipsen, and Merz; he is also an investigator on an Ipsen trial. U.D. had no disclosures to declare.

## Ethics Approval

The study was conducted in accordance with the Declaration of Helsinki and the International Conference on Harmonization Consolidated Guidelines on Good Clinical Practice, and all relevant study documents were approved by an independent ethics committee, with written informed consent obtained from all patients prior to study entry.

**CME Question**

This study showed that patients given abobotulinumtoxinA who had previously treated with other botulinum toxin type A had decreased:

- Falls
- Muscle tone
- Muscle weakness
- Pain

**Answer online at** http://me.aapmr.org

## Supporting information


**Appendix S1** Supporting Information

## Data Availability

Where patient data can be anonymized, Ipsen will share all individual participant data that underlie the results reported in this article with qualified researchers who provide a valid research question. Study documents, such as the study protocol and clinical study report, are not always available. Proposals should be submitted to datasharing@ipsen.com and will be assessed by a scientific review board. Data are available beginning 6 months and ending 5 years after publication; after this time, only raw data may be available.
