# Supplementary material for: Efficacy and Safety of AbobotulinumtoxinA for the Treatment of Hemiparesis in Adults with Lower Limb Spasticity Previously Treated With Other Botulinum Toxins: A Secondary Analysis of a Randomized Controlled Trial
Source: PM R. 2020 Mar 27;12(9):853–60. doi: 10.1002/pmrj.12348 (PMC7540573; doi:10.1002/pmrj.12348)
Supplement: Supplementary file 1 — Appendix S1 Supporting Information [file PMRJ-12-853-s001.docx]

**Supporting Information for:**

**Efficacy and safety of abobotulinumtoxinA for the treatment of hemiparesis in adults with lower limb spasticity previously treated with botulinum toxin: A secondary analysis of a randomized controlled trial**

**Contents:**

Two Supplementary Tables

**Supplementary Table 1.** Change in muscle tone in GSC and Soleus muscles at Weeks 1, 4 and 12

| **Muscle group** | **Time point** | **Parameter** | **Placebo N=26** | **AboBoNT-A 1000 U  N=29** | **AboBoNT-A 1500 U N=28** | **All aboBoNT-A  N=57** |
| --- | --- | --- | --- | --- | --- | --- |
| **GSC** | Week 1 | n | 26 | 29 | 28 | 57 |
|  |  | Mean (SD) | -0.4 (0.9) | -0.5 (0.8) | -0.6 (0.8) | -0.6 (0.8) |
|  |  | Median (range) | 0.0 (-3­­–0) | 0.0 (-3–0) | 0.0 (-3–0) | 0.0 (-3–0) |
|  | Week 4 | n | 26 | 29 | 28 | 57 |
|  |  | Mean (SD) | -0.4 (0.7) | -0.8 (1.1) | -0.9 (1.0) | -0.8 (1.1) |
|  |  | Median (range) | 0.0 (-3–0) | 0.0 (-4–0) | -1.0 (-3–1) | -1.0 (-4–1) |
|  | Week 12 | n | 25 | 28 | 26 | 54 |
|  |  | Mean (SD) | -0.2 (0.4) | -0.6 (1.0) | -0.7 (1.0) | -0.7 (1.0) |
|  |  | Median (range) | 0.0 (-1–0) | 0.0 (-3–0) | -1.0 (-4–1) | 0.0 (-4–1) |
| **Soleus** | Week 1 | n | 26 | 29 | 28 | 57 |
|  |  | Mean (SD) | -0.6 (0.8) | -0.6 (0.9) | -0.5 (0.7) | -0.6 (0.8) |
|  |  | Median (range) | -0.5 (-3–0) | 0.0 (-3–1) | 0.0 (-2–0) | 0.0 (-3–1) |
|  | Week 4 | n | 26 | 29 | 28 | 57 |
|  |  | Mean (SD) | -0.5 (0.7) | -0.7 (1.0) | -0.9 (1.0) | -0.8 (1.0) |
|  |  | Median (range) | 0.0 (-2–1) | -1.0 (-3–1) | -1.0 (-3–0) | -1.0 (-3–1) |
|  | Week 12 | n | 25 | 28 | 26 | 54 |
|  |  | Mean (SD) | -0.3 (0.9) | -0.7 (1.0) | -0.7 (0.9) | -0.7 (0.9) |
|  |  | Median (range) | 0.0 (-3–2) | -1.0 (-3–1) | -1.0 (-3–1) | -1.0 (-3–1) |

Data presented for the intention-to-treat population. Muscle tone was measured on the Modified Ashworth Scale
AboBoNT-A, abobotulinumtoxinA; GSC, gastrocnemius-soleus complex; n, number of patients who contributed data at the time point

**Supplementary Table 2.** Treatment-emergent adverse events after a single cycle of treatment in previously treated patients

|  | **Placebo N=26** | **AboBoNT-A 1000 U N=30** | **AboBoNT-A 1500 U N=28** | **All aboBoNT-A N=58** |
| --- | --- | --- | --- | --- |
| **Any TEAE** | 13 ( 50.0) [34] | 15 ( 50.0) [36] | 16 ( 57.1) [31] | 31 ( 53.4) [67] |
| **Any TEAE leading to withdrawal** | 1 ( 3.8) [1] | 2 ( 6.7) [2] | 1 ( 3.6) [1] | 3 ( 5.2) [3] |
| **Any SAEs** | 1 ( 3.8) [1] | 2 ( 6.7) [2] | 1 ( 3.6) [1] | 3 ( 5.2) [3] |
| **TEAEs by primary system organ class and preferred term** |  |  |  |  |
| **Musculoskeletal and connective tissue disorders** | 6 ( 23.1) [11] | 8 ( 26.7) [11] | 10 ( 35.7) [10] | 18 ( 31) [21] |
| Muscular weakness | 2 ( 7.7) [2] | 2 ( 6.7) [2] | 4 ( 14.3) [4] | 6 ( 10.3) [6] |
| Pain in extremity | 2 ( 7.7) [2] | 1 ( 3.3) [1] | 2 ( 7.1) [2] | 3 ( 5.2) [3] |
| Myalgia | 1 ( 3.8) [1] | 0 | 2 ( 7.1) [2] | 2 ( 3.4) [2] |
| Arthralgia | 1 ( 3.8) [1] | 3 ( 10) [3] | 0 | 3 ( 5.2) [3] |
| Back pain | 1 ( 3.8) [1] | 2 ( 6.7) [2] | 0 | 2 ( 3.4) [2] |
| **Injury, poisoning and procedural complications** | 1 ( 3.8) [7] | 4 ( 13.3) [8] | 3 ( 10.7) [4] | 7 ( 12.1) [12] |
| Fall | 1 ( 3.8) [5] | 4 ( 13.3) [5] | 3 ( 10.7) [4] | 7 ( 12.1) [9] |
| **Infections and infestations** | 2 ( 7.7) [2] | 2 ( 6.7) [2] | 3 ( 10.7) [4] | 5 ( 8.6) [6] |
| Nasopharyngitis | 1 ( 3.8) [1] | 0 | 2 ( 7.1) [2] | 2 ( 3.4) [2] |
| **General disorders and administration site conditions** | 0 | 3 ( 10) [3] | 2 ( 7.1) [2] | 5 ( 8.6) [5] |
| Fatigue | 0 | 0 | 2 ( 7.1) [2] | 2 ( 3.4) [2] |
| Influenza-like illness | 0 | 2 ( 6.7) [2] | 0 | 2 ( 3.4) [2] |
| **Psychiatric disorders** | 0 | 2 ( 6.7) [3] | 2 ( 7.1) [2] | 4 ( 6.9) [5] |
| Depression | 0 | 2 ( 6.7) [3] | 2 ( 7.1) [2] | 4 ( 6.9) [5] |
| **Gastrointestinal disorders** | 1 ( 3.8) [1] | 2 ( 6.7) [2] | 1 ( 3.6) [2] | 3 ( 5.2) [4] |
| Dysphagia | 0 | 1 ( 3.3) [1] | 1 ( 3.6) [1] | 2 ( 3.4) [2] |

Data are presented for the safety population and reported as n (%) [number of events]. Data shown are TEAEs that were reported in at least one patient who received AboBoNT-A treatment. Adverse events were coded using the Medical Dictionary for Regulatory Activities (MedDRA, version 16.0)
AboBoNT-A, abobotulinumtoxinA; TEAE, treatment-emergent adverse event; SAE, serious adverse event
